# Data from cryo-neutron phase change experiments with LH2 and LCH4

**DOI:** 10.1016/j.dib.2022.108474

**Published:** 2022-07-16

**Authors:** Kishan Bellur, Ezequiel F. Medici, Daniel S. Hussey, David L. Jacobson, Jacob LaManna, Juscelino B. Leao, Julia Scherschligt, James C Hermanson, Chang Kyoung Choi, Jeffrey S. Allen

**Affiliations:** a University of Cincinnati, Cincinnati, OH 45221, United States; b Michigan Technological University, Houghton, MI 49931, United States; c National Institute of Standards and Technology, Gaithersburg, MD 20899, United States; d University of Washington, Seattle, WA 98105, United States

**Keywords:** Liquid hydrogen, Liquid methane, Evaporation, Condensation, Thin film, Cryogenics, Neutron imaging

## Abstract

Cryogenic Propellant management is a critical roadblock to enable long term space missions. Commonly used propellants (liquid hydrogen and methane) undergo constant vaporization but there is limited knowledge on the phase change rate and its implications on long term storage stability. This is, in part, due to the inability to image the liquid-vapor mixture inside opaque metallic containers at cryogenic temperatures. Here, neutron imaging is used as a visualization technique to track the liquid-vapor interface inside Al 6061 and SS 316 test cells. The data contains first known images of steady evaporation/condensation in cryogenic propellants. The experiments were conducted at the NIST Center for Neutron Research using the BT-2 Neutron Imaging facility. The test cells were instrumented with temperature sensors and inserted into a 70-mm liquid helium cryostat before being placed into the neutron beam. Temperatures and pressures were altered to achieve condensation/evaporation and Neutron images were captured during the entire phase change process. Phase change rates were obtained through image processing. The data contains raw images and processed phase change rates along with experimental temperature and pressure. The one-of-a-kind data could be used for model validation, correlation development or serve as a benchmark for future experiments.

**Specifications Table**

**Table:** 

Subject	Chemical Engineering
Specific subject area	Liquid-vapor phase change is an interdisciplinary interfacial transport phenomena of interest in many fields of engineering. Of pertinent interest here is the applicability of liquid hydrogen/methane phase change to space sciences and cryogenics.
Type of data	Table,Image,Figure
How the data were acquired	The data was acquired at the Neutron Imaging Facility at the NIST Center for Neutron Research in Gaithersburg, MD. Temperatures were measured by Lakeshore DT-640 Si-diode sensors while pressure was monitored using Mensor (DPG 15000 and CPG 2500) sensors. Images were post processed to obtain evaporation/condensation rates.
Data format	Raw,Analyzed,Processed
Description of data collection	Cylindrical test containers of various materials (SS 316 and Al 6061) and sizes (5 mm – 30 mm) were mounted on a universal sample holder were inserted into a 70 mm liquid helium cooled cryostat and placed in the beamline. Images were captured at 10 s intervals while temperatures and pressures were captured every second. The tests were repeated for different test cells and base pressures. Tests were conducted with both hydrogen and methane as the phase change fluid.
Data source location	• Institution: Michigan Technological University• City/Town/Region: Houghton, MI• Country: United States
Data accessibility	Repository name: Data from cryo-neutron phase change experiments with LH2 and LCH4Data identification number: 10.17632/z5zc7kk76g.2Direct URL to data: http://dx.doi.org/10.17632/z5zc7kk76g.2
Related research article	K. Bellur , E. F. Médici, D. S. Hussey, D. L. Jacobson, J. LaManna, J. Leão, J. Scherschligt, J. Hermanson, C. K. Choi and J. S. Allen, “Results from neutron imaging phase change experiments with LH2 and LCH4”, Cryogenics. In Press. DOI: 10.1016/j.cryogenics.2022.103517.

**Value of the Data**

• The data contains first-of-its-kind neutron image data during steady evaporation/condensation of cryogenic propellants (liquid hydrogen and methane)
• The data is beneficial to cryogenics and space science research communities.
• The data serves as a proof-of-concept and a benchmark for future experiments.
• The data enables model validation for cryo-storage stability in long term space missions.
• The data allows for correlation development for future mission design.

## 1 Data Description

The data repository [1] contains processed and reduced data from the cryogenic neutron imaging phase change experiments conducted in the BT-2 Neutron Imaging Facility at NIST Center for Neutron Research in Gaithersburg, MD. The data is organized into folders based on different test conditions. A summary of test conditions for experiments with liquid hydrogen and liquid methane are shown in Tables 1 and 2 respectively.

**Table 1:** Summary of test conditions for liquid hydrogen experiments conducted in January.

Test cell #	Run #	Pressure (psia)	Saturation Temperature (K)	Condensation Sub-cool (K)	Evaporation Super-heat (K)
**tc1 (Conical cell)**	*Run 1*	17.9	21	20.8/20.6	22/23
	*Run 2*	17.9	21	19	25
	*Run 3*	13.8	19.9	19	21/22
	*Run 4*	13.8	19.9	18.5	25
	*Run 5*	28.6	23	22.7/22.5	N/A
	*Run 6*	20.6	21.6	N/A	22.5
	*Run 7*	25.4	22.5	21.5	N/A
	*Run 8*	25.4	22.5	N/A	28

**tc2 (10mm SS)**	*Run 1*	17.9	21	19.9	22
	*Run 2*	13.8	19.9	18.8	21
	*Run 3*	28.6	23	21.9	23.5
	*Run 4*	28.6	23	22.5	24/25/27
	*Run 5*	28.6	23	22.5	28

**tc3 (30mm Al)**	*Run 1*	17.9	21	20/20.7	22/24/25

**tc3 (10mm Al)**	*Run 1*	17.9	21	19	23
	*Run 2*	13.8	19.9	17	22
	*Run 3*	28.6	23	20	26
	*Run 4*	28.6	23	20.5	26

**Table 2:** Summary of test conditions for liquid methane experiments conducted in September.

Test cell #	Run #	Temperature (K)	Saturation Pressure (psia)	Condensation pressure (psia)	Evaporation pressure (psia)
**tc1 (10 mm Al)**	*Run 1*	121	30	30.2	Could not be held constant
	*Run 2*	115.4	20	20.4	17.6
	*Run 3*	111.9	15	15.7	12.7
	*Run 4*	116.8	22	22.5	20.2
	*Run 5*	121	30	31.2	27.1
	*Run 6*	114.2	18	18.6	17

The corresponding data is organized into a folder structure, as follows:

• **Jan***◊ hydrogen data*
  ○ **jan_tcX_runY**
    ■ **jan_tcX_runY_exp_jpg***◊ neutron images*
    ■ jan_tcX_runY_exp_daq.csv *◊ daq data*
    ■ jan_tcX_runY_exp_img.csv *◊ tabulated data for each image*
    ■ jan_tcX_runY_exp_img_rate.csv *◊ evaporation/condensation rate(s)*
    ■ jan_tcX_runY_exp_press_heat.png *◊ plot of pressure and heater power*
    ■ jan_tcX_runY_exp_temp.png *◊ plot of temperature*
    ■ jan_tcX_runY_exp_vol.png *◊ plot of liquid volume*
    ■ jan_tcX_runY_exp_thick.png *◊ plot of film thickness*
• **Sept***◊ methane data*
  ○ **sept_tc1_runY**
    ■ **sept_tc1_runY_exp_jpg***◊ neutron images*
    ■ sept_tc1_runY_exp_daq.csv *◊ daq data*
    ■ sept_tc1_runY_exp_img.csv *◊ tabulated data for each image*
    ■ sept_tc1_runY_exp_img_rate.csv *◊ evaporation/condensation rate(s)*
    ■ sept_tc1_runY_exp_press_heat.png *◊ plot of pressure and heater power*
    ■ sept_tc1_runY_exp_temp.png *◊ plot of temperature*
    ■ sept_tc1_runY_exp_vol.png *◊ plot of liquid volume*
    ■ sept_tc1_runY_exp_thick.png *◊ plot of film thickness*

Here, **X** represents test cell # and **Y** represents test run #. . Each test contains a **folder of neutron images**, 3 csv files and 4 png files. These are detailed below:

• The images in the *_jpg folder contain an image #, elapsed time and average pressure and outer wall temperature measured during the image integration time of 10s. The images are median filtered, adjusted for contrast and embedded with test condition data. Unprocessed neutron images are available on request.
• The *daq.csv files contain the data measured by the DAQ: 3 outer wall temperatures (s2-s4), helium vapor temperature (s1), heater temperature (htr), sample holder temperature (stick), pressure (p_kpa) and heater power (htr_pow) as a function of time. Temperatures are in K, time in s, pressure in kPa and heater power is a % where 100% = 5W.
• The *img.csv files tabulate the avg temperatures (s1-s4, htr, stick), vapor pressure (p_kpa), heater power (htr), volume (vol_*), film thickness (thickness) and location of apex (apex). Temperatures are in K, time in s, pressure in kPa, volume in mm^3^, thickness in µm, apex in pixel location value and heater power is a % where 100% = 5W. Most files have volume computed from both interface tracking (vol_it) and optical density (vol_od). Some files also contain additional information such as the attenuation coefficient (µ) and beam hardening factor (β) for either the liquid or the vapor.
• The *img_rate.csv files contain data on the evaporation/condensation rates computed from the vol vs time data. The image window for which the rate was computed, the mean and standard deviation in the pressure and temperature during the image window are also provided in addition to the computed rate.
• The *.png plots utilize data from the csv files described above and are provided only for quick, convenient visualization. The user is encouraged create custom high-resolution visuals from the csv data provided.

## 2 Experimental Design, Materials and Methods

The high neutron cross section of hydrogen in comparison with metals allows for the ability to visualize a hydrogenated fluid inside an opaque metallic container. In this study, we use the BT-2 Neutron Imaging Facility at NIST Center for Neutron Research in Gaithersburg, MD, USA to visualize the evaporation/condensation process in hydrogen and methane at temperatures as low as 18K and 100K respectively. The BT-2 beam line uses thermalized neutrons with an energy of 25 meV. The low energy does not significantly alter evaporation/condensation and the increase in temperature of the fluid is estimated to be < 1 µK. Temperatures, pressures, and neutron images are recorded during the phase change process. The images are then processed to calculate the phase change rates and film thicknesses using a combination of ImageJ and MATLAB. The motivation behind the experiments [2], neutron imaging [3], and cryogenic heat transfer [4] are described elsewhere and the experimental design is summarized below.

### 2.1 Setup

**Fig. 1:**
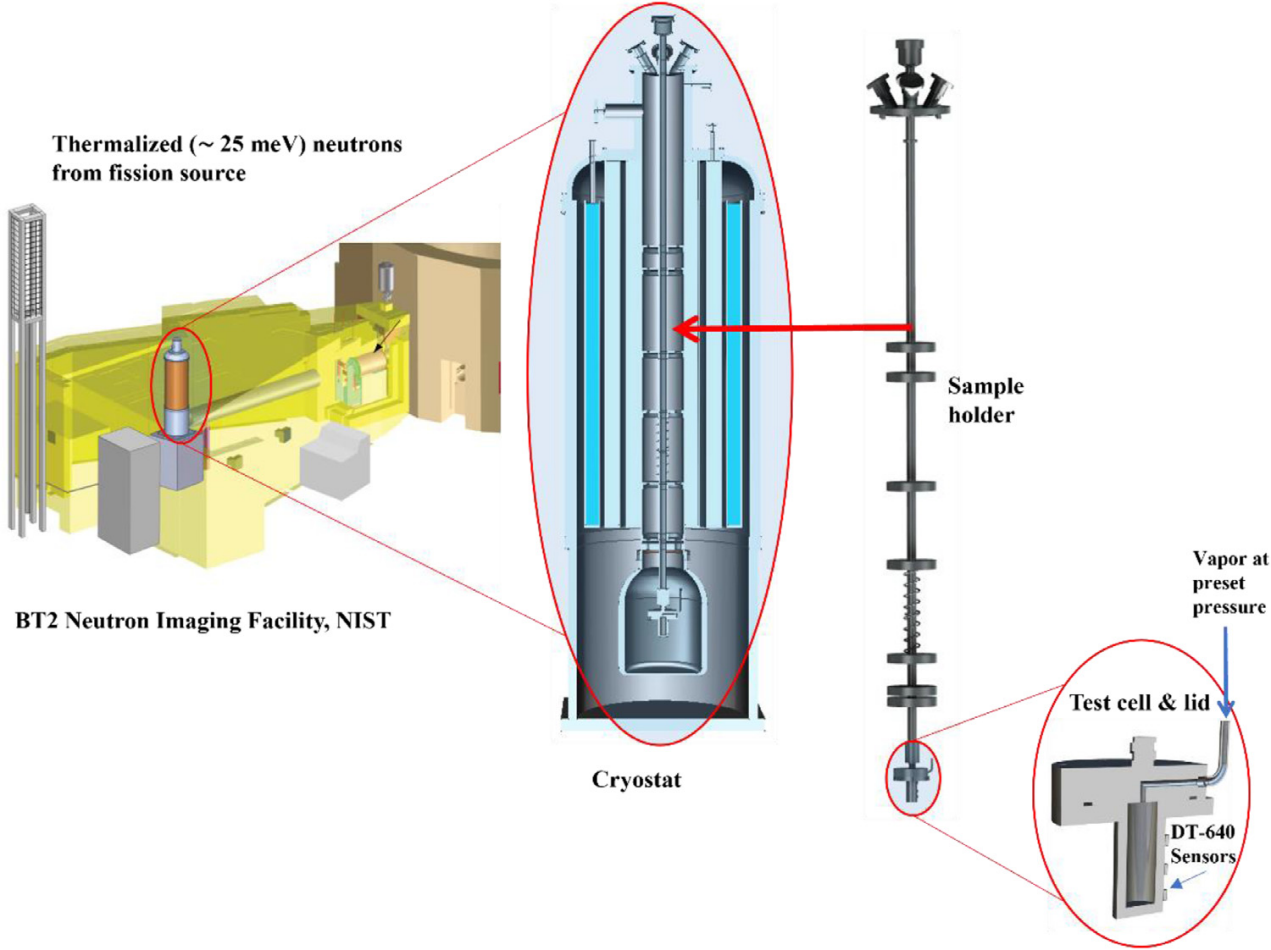
Test cells instrumented with DT-640 sensors are mounted on the sample holder using a universal lid with a vapor feed port. The sample holder is inserted into a 70 mm liquid helium pumped cryostat and placed in the Neutron Imaging Facility.

Cylindrical test cells of various sizes (5 – 30 mm) are fabricated from SS 316 and Al 6061 stock material. A universal lid is fabricated from SS 316 such that it could be used with all test cells. The lid is attached to the test cell using an indium seal. Several Lakeshore DT-640 Si-diode temperature sensors (S1-S4) are mounted on the test cell at various locations [5]. The test cell/lid assembly is attached to a sample holder and then inserted into a 70 mm bore pumped liquid helium cryostat (Fig. 1).

### 2.2 Imaging

**Fig. 2:**
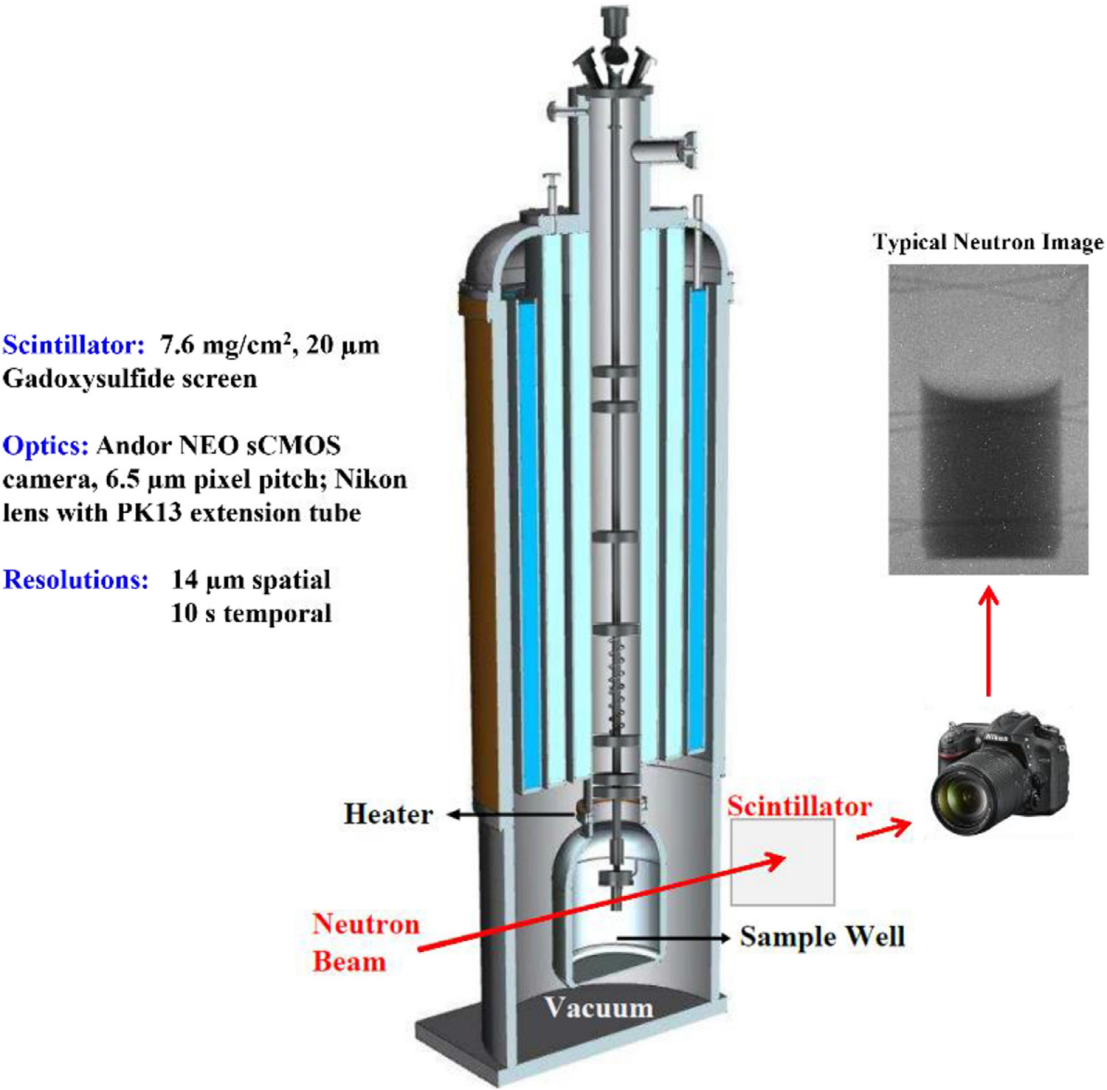
The cryostat is placed in the beamline such that the neutrons pass through the test cell and strike the scentillator placed downstream. A sCMOS camera is used to capture a typical neutron image where the dark region represents liquid.

The cryostat with inserted sample holder is placed in the beamline of the BT-2 Neutron Imaging Facility such that the center of the beam passes through the test cell (Fig. 2). Thermalized neutrons with an energy of 25 meV pass through the cryostat and strike the scintillator placed downstream. A 20 µm Gadoxysulfide film is used as a scintillator and an Andor NEO sCMOS camera with a PK-13 extension tube is used to focus the neutron image. To allow for sufficient contrast, the images are captured every 10 seconds.

### 2.3 Operation

**Fig. 3:**
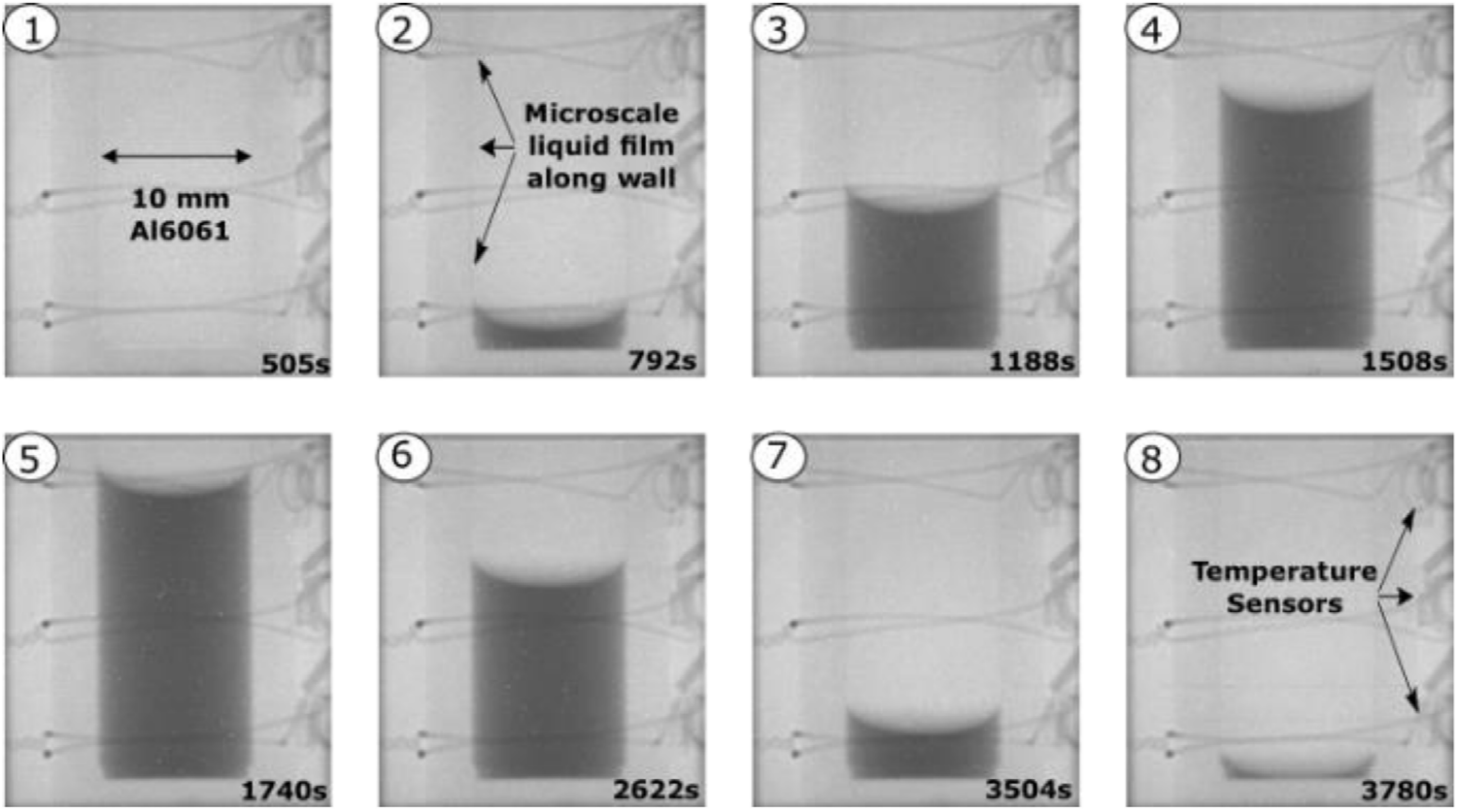
A typical condensation (1-4) and evaporation (5-8) test with hydrogen where the liquid fill level varies with time.

The cryostat, test cell and vapor lines are purged with helium gas and evacuated to ensure purity prior to introducing the propellants. A small amount of helium gas is added to the cryostat sample well to aid in thermal transport after the last evacuation. Cryogen vapor is introduced at a constant preset pressure and the heater temperature is decreased below the saturation condition to initiate condensation. After sufficient rise in the liquid fill level, the temperature is increased above saturation to initiate evaporation. During the entire process images are captured every 10 s while the temperatures (S1-S4, heater, etc) and pressures (manifold and sample) are captured every second. Fig. 3 shows a typical test where images 1-4 shows condensation and images 5-8 shows subsequent evaporation. The process is repeated for different pressures and test cells. In the methane experiments, the temperature was held constant and the pressure was altered to achieve phase change.

### 2.4 Post-processing

To correlate the imaging data with DAQ data (temperatures and pressures), the avg temperatures and pressures for the 10 s duration of each image is extracted and embedded directly into the image along with the corresponding time stamp. Background radiation noise is removed using a median filter and enhanced for better contrast. The evaporation/condensation rates are obtained by picking a imaging window and then using edge detection based interface tracking and optical density transformation (detailed in a separate manuscript [3]) to estimate the rate of condensation/evaporation.

## Ethics Statements

The work described here does not involve human subjects, animal experiments or social media platforms. All funding sources and stakeholders are acknowledged, and the data is not published elsewhere.

## CRediT Author Statement

**Kishan Bellur:** Investigation, Methodology, Formal Analysis, Writing – original draft; **Ezequiel F. Medici:** Investigation, Methodology; **Daniel S. Hussey:** Methodology, Resources, Software, Visualization, Writing – review & editing; **David L. Jacobson:** Resources, Software; **Jacob LaManna:** Resources; **Juscelino B. Leao:** Resources; **Julia Scherschligt:** Resources; **James C Hermanson:** Conceptualization, Writing – review & editing; **Chang Kyoung Choi:** Conceptualization, Supervision; **Jeffrey S. Allen:** Conceptualization, Supervision, Project administration, Writing – review & editing.

## Declaration of Competing Interest

The authors declare that they have no known competing financial interests or personal relationships that could have appeared to influence the work reported in this paper.

## Data Availability

Data from cryo-neutron phase change experiments with LH2 and LCH4 (Original data) (Mendeley Data).
